# Different morphologies of super-balls obtained to form photonic crystals of cholesteryl benzoate liquid crystals

**DOI:** 10.1039/d4na00431k

**Published:** 2024-09-06

**Authors:** Edina Rusen, Alexandra Mocanu, Adrian Dinescu, Adina Boldeiu, Cosmin Romanitan, Sergiu Iordanescu, Martino Aldrigo, Raluca Somoghi, Raul Mitran, Adi Ghebaur

**Affiliations:** a Faculty of Chemical Engineering and Biotechnologies, National University of Science and Technology Politehnica Bucharest 1-7 Gh. Polizu Street Bucharest 011061 Romania edina_rusen@yahoo.com; b National Institute for Research and Development in Microtechnologies—IMT Bucharest 126A Erou Iancu Nicolae Street 077190 Bucharest Romania; c Oil-Gas University of Ploiesti 39 B-dul Bucuresti 100520 Ploiesti Romania; d The National Institute for Research & Development in Chemistry and Petrochemistry 202, Spl. Independentei 060021 Bucharest Romania; e “Ilie Murgulescu” Institute of Physical Chemistry, Romanian Academy 202 Splaiul Indepedentei Bucharest 060021 Romania; f Advanced Polymer Materials Group, Faculty of Chemical Engineering and Biotechnology Romania

## Abstract

The current study highlights the synthesis and characterization of some nanocomposite materials formed by polymer particles and liquid crystals. The liquid crystals used were cholesteryl benzoate (CLB), and the particles were synthesized by emulsion polymerization in the absence of the emulsifier. Through SEM and DLS analysis, the synthesis of particles of the same size was emphasized, and the amount of CLB showed no influence on these parameters. The lack of signal for CLB in the case of DSC and XRD analyses for the sample with the smallest amount of liquid crystal is attributed to the detection limits of the devices. To complete the surface characterization of the particles, XPS analysis was performed. Through XPS it was underlined that in the case of the smallest amount and the largest amount of CLB, respectively, it is encapsulated in the polymer particles, unlike the case of the average amount used, in which core–shell type morphologies have been obtained. For the electrical characterization of the samples, a Vector Network Analyzer (VNA) connected to two rectangular X-band (*i.e.*, 8.2–12.4 GHz) waveguides through coaxial cables was used.

## Abstract 2

The current study highlights the synthesis and characterization of some nanocomposite materials formed by polymer particles and liquid crystals.

## Graphical abstract

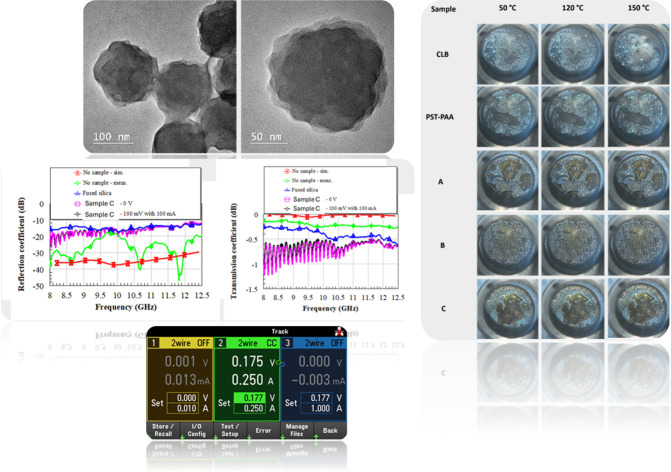

## 1. Introduction

**Scheme 1:**
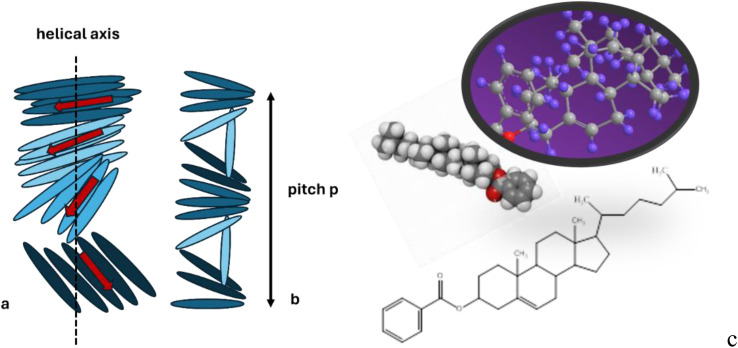
The helicoidal structure of the CLC: (a) the twist in the arrangement of rod-like molecules is shown with the help of the rotating red arrow; (b) a complete revolution of the arrow along the helicoidal axis occurs over a length equal to the pitch; (c) chemical structure of CLB.^2^

The cholesteric phase, also known as the chiral nematic liquid crystal (CLC) phase, is primarily made up of nematic mesogenic molecules, which are in a state of matter between solid and liquid. These molecules have a chiral center, which generates intermolecular forces that encourage molecular alignment at a small angle to one another.^1,2^ As a result, a structure can be seen as a collection of extremely thin, two-dimensional, nematic-like layers, with each sheet's direction twisted with the sheets above and below (Scheme 1a). The director axis typically varies periodically, this variation (the distance over which a full rotation of 360° is completed) being known as the pitch, p. This pitch determines the wavelength of light which is reflected (Bragg reflection) (Scheme 1b).^3–6^

Most nanomaterials are known to thermodynamically exhibit achiral crystal structures. To obtain chiral nanomaterials, the most straightforward method is the enantioselective synthesis of chiral nanocrystals with mirror-asymmetric geometry *via* seed-mediated colloidal growth.^7–9^

Cholesteryl benzoate (CLB) (Scheme 1c), which exhibits CLC, is well known as the first liquid crystalline compound found by Reinitzer in 1888.^10^ Taking into account the polymer's physical properties, the polymer film can stabilize the mesogenic orientation of the CLC,^11^ obtaining in this way chiral nanomaterials. Thus, polymer-dispersed liquid crystals (PDLCs) are composite materials in which a liquid crystalline material is dispersed within a polymer matrix to form micron-sized droplets. PDLC films have a variety of electro-optical applications ranging from window shutters to projection displays, and even chemical sensors.^12^ The thermal and mechanical stability of thermo-optical devices is improved by dispersing a liquid crystalline material within a polymer matrix.^13,14^ PDLC can be produced using methods such as encapsulation and phase separation.^15–18^ Encapsulation is when the liquid crystal is surrounded by a layer of polymer. Six different synthesis pathways—including interfacial polymerization, *in situ* polymerization, complex coacervation, solvent evaporation, microfluidics, and polymerization of reactive mesogens—are presented in the literature for the encapsulation technique in which the liquid crystals are entrapped in a layer of polymer. These pathways take into account the specific physicochemical properties of CLCs.^1,19–21^

Polymers play an important role in the development of materials for photonics.^22^ Polymers are relatively inexpensive, can be functionalized to achieve required optical, electronic, or mechanical properties, and have demonstrated compatibility with various patterning methods. Also, they can be used as materials for photonic applications in several ways. First, polymers can inherently exhibit useful optical properties such as electroluminescence, photoluminescence, or nonlinear optical properties.^23^ Second, polymers can act as matrices for optically active species, *e.g.* for dyes, liquid crystals (LCs), quantum dots, or metal nanoparticles.^22^ Third, polymers possessing topographic and/or compositional patterns can coherently scatter light.^24^ Finally, polymer templates are routinely used for producing photonic materials.^25^

At the same time, polymer-dispersed liquid crystals can be profitably exploited as coating materials for different high-frequency applications. In this respect, the so-called X band (8.2–12.4 GHz) is of utmost importance for radars, terrestrial and space communications, traffic light motion sensors, and the RF sources of particle accelerators. All the previous systems are prone to electromagnetic interference (EMI) issues that can be tackled by using various lightweight materials for EMI shielding purposes, such as conducting polymers, graphene, carbon nanotubes, and other nanomaterials.^26^ Hence, the microwave characterization of polymeric coatings through different methods, *e.g.*, by means of standard waveguides, provides significant insight into their suitability as a material offering either EM transparency or EMI shielding properties.

Knowing that CLC and polymer photonic crystals (PPC) reflect light under the same mechanism described by Bragg's law, the current study emphasizes the configuration of a system formed by the two components to intensify the obtained optical signals. On the other hand, the studies undertaken by our team^27,28^ highlighted the mechanism of generation of polymeric photonic crystals in which CL is in dispersed form, being obtained through emulsion polymerization in the absence of surfactant.^29–33^

The present work emphasizes the synthesis and characterization of a new material consisting of CLB and poly(styrene-*co*-acrylic acid) (PST-PAA) copolymer. The polymerization process consisted in employing emulsion polymerization in the absence of surfactant, highlighting the morphology of the obtained particles, the influence of different concentrations of CLB on the polymerization reaction and the possible applications of these systems.

The study is highly intriguing, bringing new outcomes in the literature for the first time to our knowledge, giving a novel insight on achieving two distinct morphologies based on the concentration of CLB utilized within this field.

## 2. Materials and methods

### 2.1. Materials

Styrene (ST) (Sigma-Aldrich) and acrylic acid (AA) (Sigma-Aldrich) were purified through vacuum distillation. Potassium persulfate (K_2_S_2_O_8_) (KPS) (Merck) was recrystallized from an ethanol/water mixture and then vacuum-dried. Liquid crystal cholesteryl benzoate (CLB) (Sigma-Aldrich) was used as received.

### 2.2. Methods

#### 2.2.1. Synthesis of the polymer liquid crystal systems (A, B and C)

Three samples denoted A, B and C were obtained by soap-free aqueous emulsion polymerization. 1.3 mL of ST, 0.25 mL of AA and different amounts of CLB (0.1 g (A), 0.2 g (B), and 0.3 g (C)) were added to 20 mL of distilled water with 13 mg of KPS. The polymerization reactions were nitrogen purged and then maintained for 4 h at 80 °C under continuous stirring at 350 rpm.

#### 2.2.2. Synthesis of polymeric films based on PST-PAA-CLB

The final emulsions of A, B and C were dialyzed for 7 days in distilled water using cellulose dialysis membranes (molecular weight cutoff, 12 000–14 000 Da) to remove the unreacted monomers and initiator. The water used for the dialysis procedure was changed every 12 hours. The film was obtained by gravitational sedimentation on glass substrates and dried at 30 °C for 8 h.

### 2.3. Characterization

The morphological and structural characterization of the particles was carried out with a Nova NanoSEM 630 Scanning Electron Microscope (FEI Company, Hillsboro, OR, USA) at an acceleration voltage of 10 kV.

The particle size measurement was conducted through dynamic light scattering (DLS) and zeta potential was obtained in water on Nano ZS ZEN3600 Zetasizer (Malvern Instruments, Malvern, UK) equipment.

Differential scanning calorimetry (DSC) measurements were performed using a Mettler Toledo DSC 3 calorimeter, at a scanning rate of 10 °C min^−1^, under an 80 mL min^−1^ nitrogen flow. Two heating–cooling cycles were performed between −20 and 160 °C. Transition temperatures were computed as onset temperatures, while glass transition temperatures (*T*_g_) were determined as midpoint temperatures. *In situ* optical microscopy (OM) was performed using an optical microscope equipped with an Olympus SC50 digital camera, at a frame rate of 1 image per min.

The molecular weights of the obtained polymers were analyzed using a PL-GPC 50 Integrated GPC/SEC System (Agilent Technologies) with a 1 mL min^−1^ tetrahydrofuran (THF) flow rate and a column oven temperature of 30 °C using polystyrene as a standard and a refractive index detector.

X-ray diffraction (XRD) investigations were carried out using a 9 kW Rigaku SmartLab diffractometer with a CuKα1 source (*λ* = 0.15406 nm). For the CLB powder, it was used for a *θ*/2*θ* radial scan, while for the other samples deposited on fused silica, a grazing incidence geometry was used, to reduce the signal from the substrate.

The morphologies of polymer particles were investigated using an FE-SEM (field emission-scanning electron microscope – RAITH e_Line), at 10 kV acceleration voltage. The samples were sputtered with a thin layer of gold, before imaging.

The polymer particles were also evaluated using transmission electron microscopy (TEM) (Tecnai G2 F20 TWIN CryoTEM, FEI Company), at 300 kV acceleration voltage, at a 1 Å resolution. A small drop of a well-dispersed sample was put on a carbon film copper grid, dried, and then visualized on TEM.

## 3. Results and discussion

**Fig. 1:**
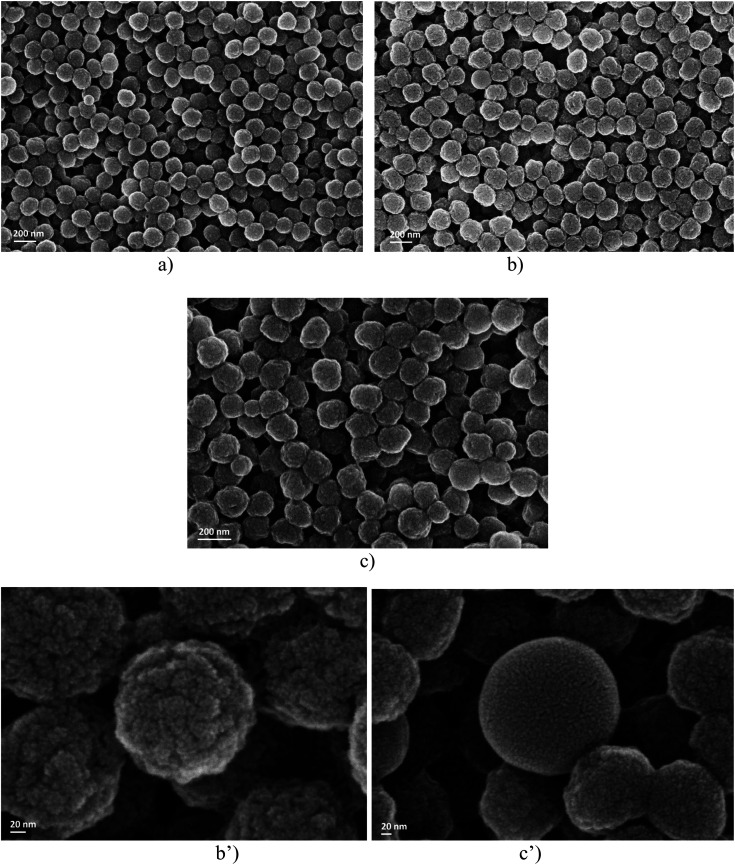
SEM analysis for samples A (a), B (b) and C (c), respectively, and the details of samples B (b′) and C (c′).

In order to obtain evidence for the influence of the CLB concentration on the soap-free emulsion polymerization reaction, the first characterization method consisted in examining the morphology of the particles using the SEM technique (Fig. 1).

Examining the dimensional aspect, it is observed that the particles obtained from emulsions A, B, and C show little difference in size. However, since only a single area of the sample was analyzed using the SEM technique, any conclusion drawn would be purely subjective.

What is rather interesting is the fact that the surfaces of the polymer particles in the case of sample B (Fig. 1b′) are rough, unlike sample C (Fig. 1c′) which seems to be much smoother, as shown by the detailed micrographs obtained by SEM analysis. The notable difference lies in the amount of CLB used during polymerization. In all three scenarios, the issue of CLB localization within the particles is brought up.

**Fig. 2:**
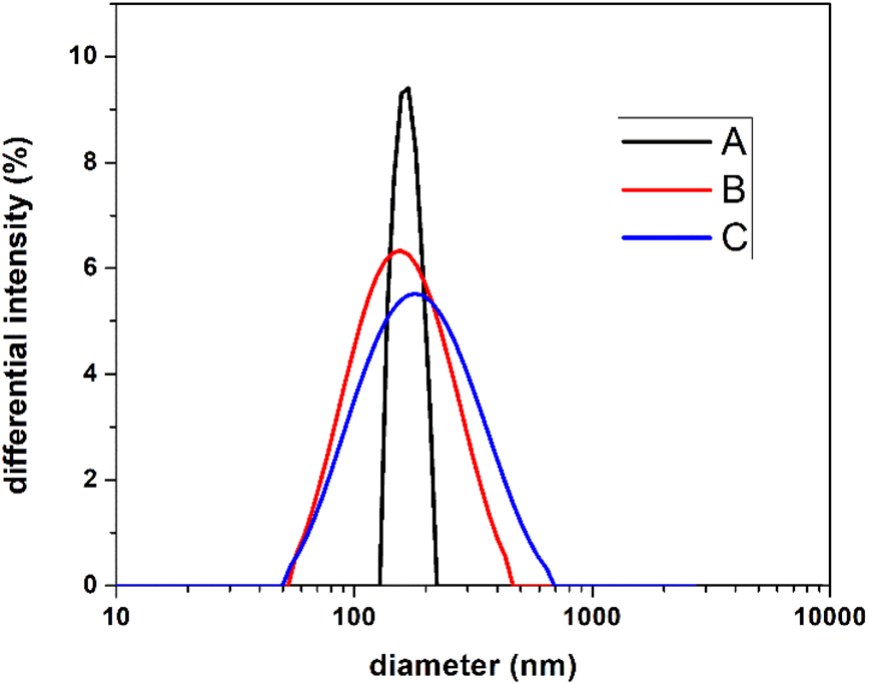
DLS analysis for samples A, B and C.

Thus, for a much more objective interpretation of our results, the size of the studied particles was investigated by the DLS technique, which analyzes the sample as a whole. Fig. 2 shows the dimensional distribution of the three analyzed samples, obtaining a medium-size diameter value of 168 nm for sample A, 141 nm for sample B, and 162 nm for sample C. No notable differences are observed regarding the average size of the particles. What is different is the dimensional distribution, a narrow distribution in the case of A, compared to B and C samples, respectively. This fact can be attributed to the nucleation mechanisms, and possible chain transfer reaction, noticeable in our previous works.^27,28^ For information on the stability of the emulsions, zeta potential measurements were performed, their values being −45 mV, −49 mV, and −52.6 mV for samples A, B, and C, respectively. In all cases, the values of zeta potential indicate moderate stability of the emulsions.^34^

**Fig. 3:**
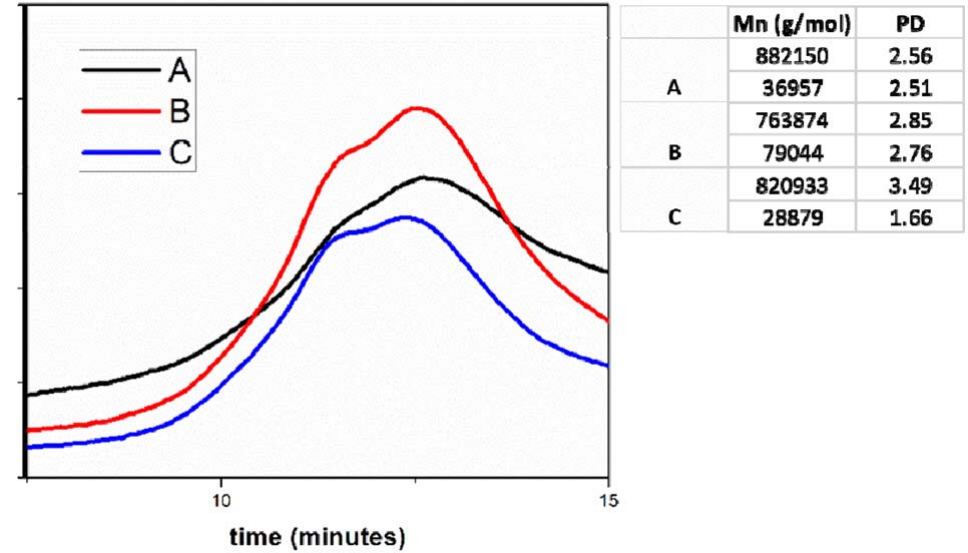
GPC analysis for samples A, B and C.

To highlight the possible generation mechanism of the particles in the soap-free emulsion polymerization and the chain transfer reactions, respectively, the next step in our study consists in the GPC analysis of the A, B and C samples. It is noticeable in Fig. 3 that the molecular weight values do not differ significantly, and the polydispersity index values (PD) are reasonably close. These observations can't explain the different polymerization mechanisms, depending on the CLB concentration. As preliminary information, we can conclude that CLB concentration does not influence the polymerization mechanism, in contrast to our previous studies.^27,28^

**Fig. 4:**
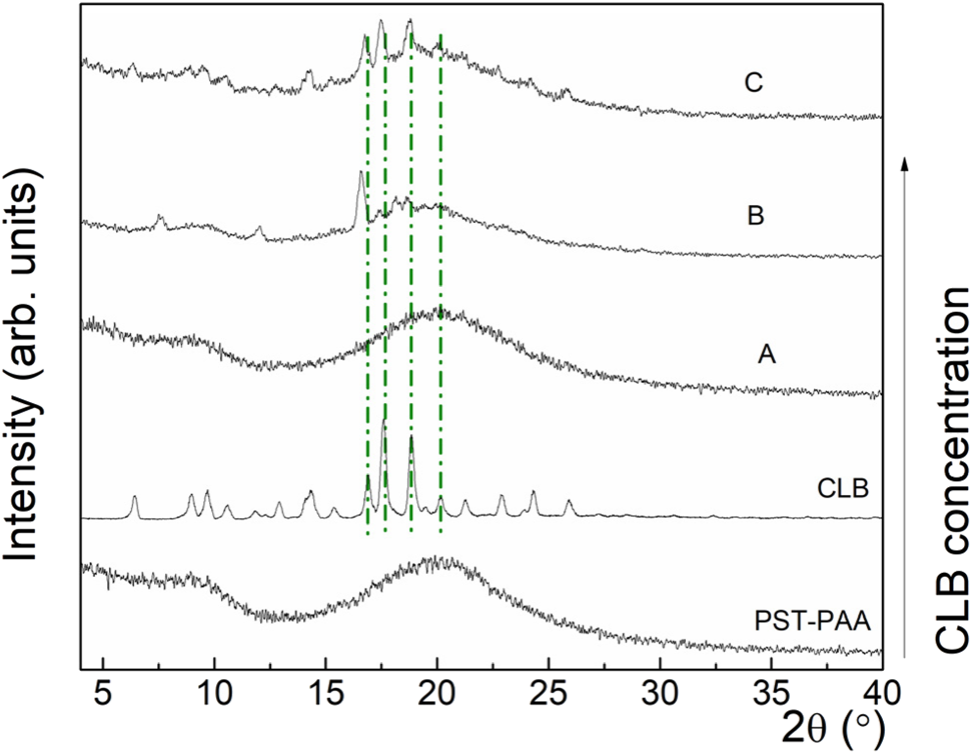
XRD patterns for PST-PAA, CLB, and samples A, B and C.

To obtain evidence for the co-existence of different CLB concentrations with the polymers, XRD analysis was used. Thus, Fig. 4 shows the XRD patterns for PST-PAA and CLB, as well as for the nanocomposites at different CLB concentrations.

For PST-PAA, the presence of two broad features, centered at ∼9° and ∼20°, characteristic of amorphous materials, can be observed. In the case of commercial CLB, a set of diffraction peaks can be observed, at 2*θ* = 6.48, 8.93, 9.69, 10.54, 11.86, 12.90, 14.32, 15.36, 16.88, 17.63, 18.86, 20.09, 21.32, 22.93, 24.34, and 25.95°. Further, at low content of CLB in polymer nanocomposites (sample A), no characteristic diffraction peaks of CLB were observed by XRD. Upon increasing the CLB concentration, in sample B characteristic diffraction peaks of CLB can be observed at 2*θ* = 7.56°, 12.06°, 16.60°, 17.26°, 18.11° and 18.86°. Finally, at the highest concentration of CLB, other diffraction peaks occurred at 2*θ* = 8.82°, 9.46°, 10.53°, 22.82°, 24.16°, and 25.81°. At the same time, the diffraction peaks from 17.49°, 18.84° and 20.02° become narrower, showing the increase of the mean crystallite size with increasing the CLB concentration.

**Fig. 5:**
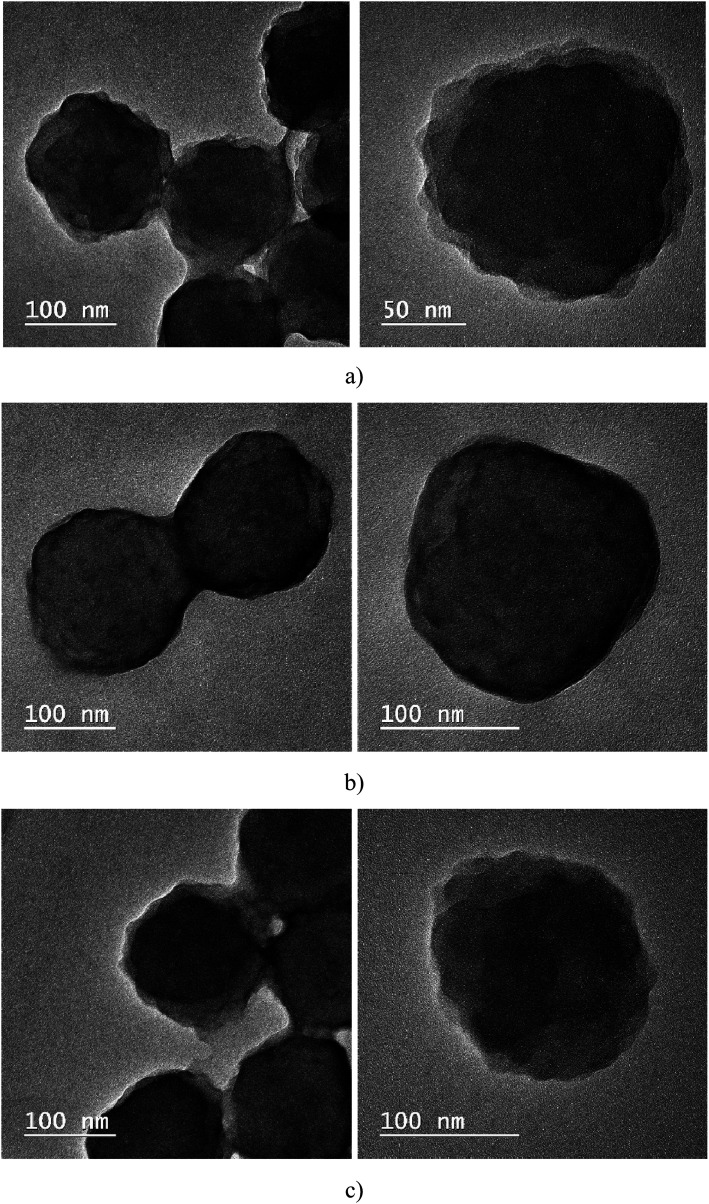
TEM images for the samples A (a), B (b) and C (c).

It seems that in sample A, CLB is missing; thus, the next step consisted in investigating the samples using the TEM technique. Through this investigation method, it is possible to observe where the CLB is located; it can be encapsulated in the polymer particle (the most common case) or form a shell around the polymer particle. Analyzing Fig. 5, it is noted that in cases A and C, CLB seems to be encapsulated, unlike sample B, where it is around the particle, and forms a shell around the polymer.

**Fig. 6:**
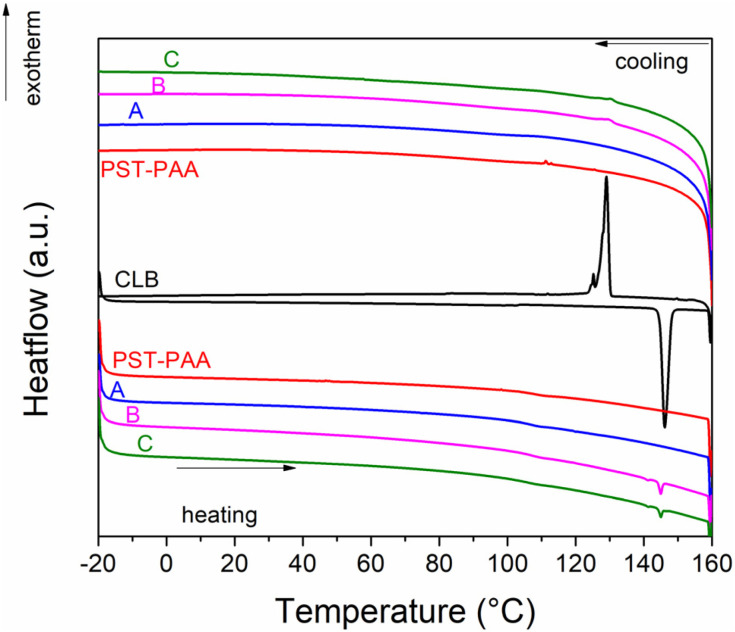
DSC analysis of CLB, PST-PAA and composite samples.

DSC analyses were carried out on the liquid crystal, polymer matrix and composite samples (Fig. 6). The CLB liquid crystal exhibits a reversible endothermic transition on heating at 144.5 °C, associated with the melting of the sample. A freezing point of 130.2 °C was noticed for this sample. The PST-PAA copolymer has a glass transition temperature of 106.7 °C during the heating run. The same glass transition temperature can be noticed for all 3 composite samples. Sample A does not show the melting of CLB, indicating that no separate liquid crystal phase is present in this sample. The melting and freezing of CLB can be noticed for samples B and C, with no significant changes in transition temperature with respect to the pristine liquid crystal. The CLB content was computed at 1.6% and 1.3% wt for samples B and C, based on their heat of fusion values.

**Fig. 7:**
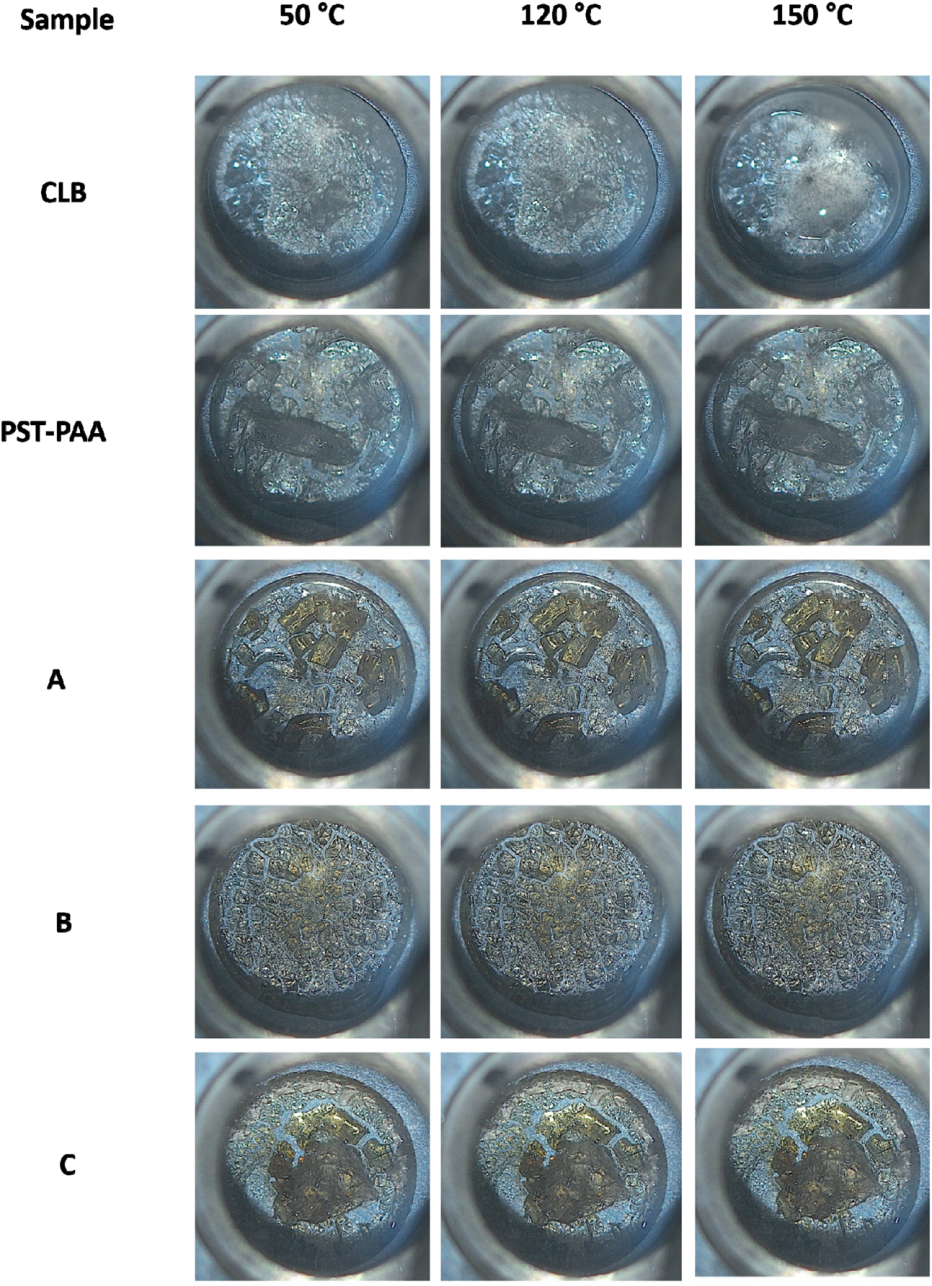
Microscopy images taken at 50 °C, 120 °C and 150 °C for the CLB, PST-PAA, and composite samples.

High-temperature optical microscopy images show that CLB is solid at 120 °C and liquid at 150 °C (Fig. 7). The CLB melting starts at 144–145 °C, in accordance with the DSC analysis. No significant changes can be noticed for the PST-PAA polymer or composite samples upon heating from 120 to 150 °C, indicating that the CLB is not present as a separate phase.

**Fig. 8:**
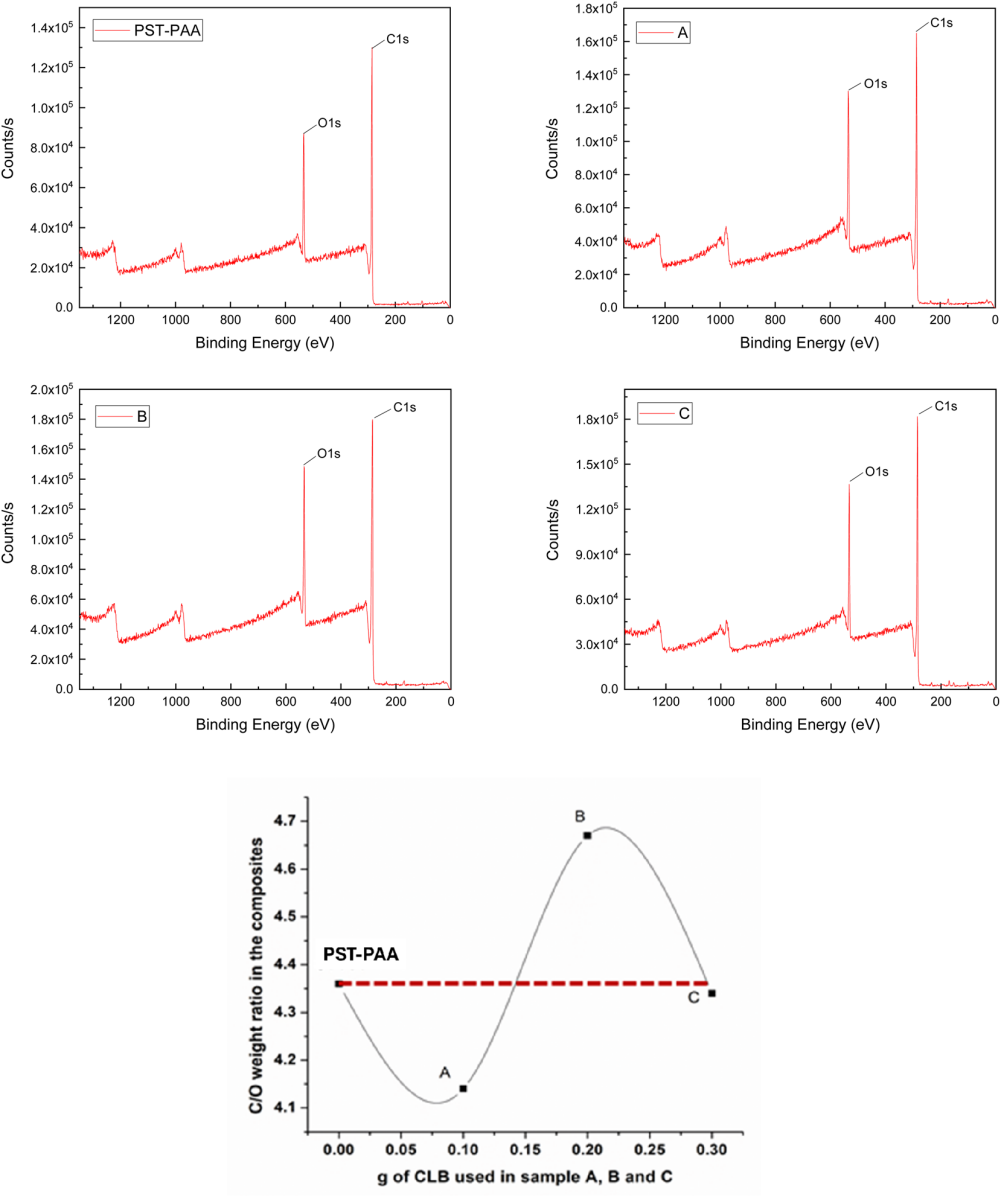
XPS results and dependence of the C/O weight ratio *versus* the CLB quantity used.

After the XRD and DSC analyses, the existence of CLB was highlighted in samples B and C, unlike sample A, where it seems to be missing. However, the lack of CLB in sample A is unjustified, as it can be explained as being below the detection limit of these devices. To elucidate the localization of CLB within the composite materials, XPS analysis was employed on the surface of the synthesized materials. For a better understanding of the results, Table 1 presents the atomic% of C 1s and O 1s respectively as well as the C/O atomic ratio, while Fig. 8 indicates the intensity of the signal for each binding energy for the specific elements identified in all samples, as well as the dependence of the C/O weight ratio *versus* the CLB quantity used.

**Table 1:** The atomic% of C 1s and O 1s, respectively, and C/O atomic ratio

Sample code	Atomic%		C/O ratio
	C 1s	O 1s	
PST-PAA	81.34	18.66	4.36
A	79.83	19.26	4.14
B	81.76	17.51	4.66
C	80.66	18.42	4.37

Knowing that the C/O weight ratio in PST-PAA, determined by XPS analysis, is 4.36, and for CLB it is 12.75, we can estimate where CLB is located in these samples, depending on the ratio obtained from XPS analysis (see Table 1). Thus, in the case of sample A, the ratio is 4.14, much lower than that of PST-PAA. This behavior can be explained by the encapsulation of CLB within the polymer particles, as opposed to sample B, where CLB is located around the particles. This is supported by the higher C/O ratio of 4.66 and the increased intensity of the O 1s peak (1.48 × 10^5^) in sample B compared to the blank sample, which registered a peak intensity of 8.58 × 10^4^. For the other CLB-based samples, the O 1s peak intensity was 1.29 × 10^5^ for sample A and 1.34 × 10^5^ for sample C. From these data, for sample C it is evident that CLB is inside the particles. In conclusion, it is possible to show that using different quantities of CLB, the morphologies of the particles are different, consistent with the results of TEM analysis.

### 3.1. Applications

**Fig. 9:**
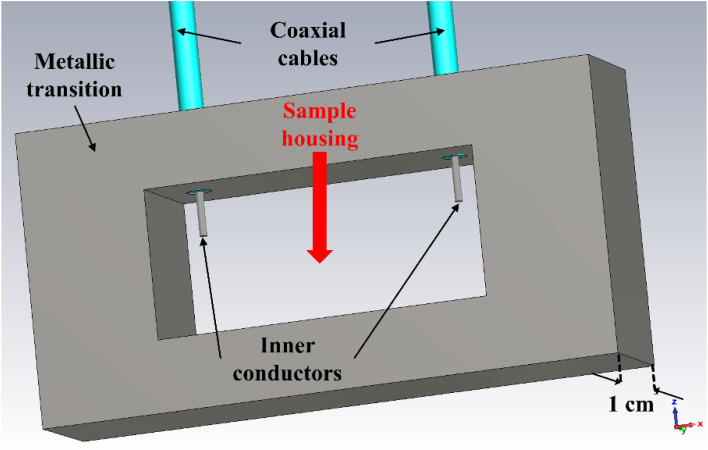
EM layout of the metallic transition used to apply an external DC bias voltage to a DUT placed inside the X-band waveguides.

For the electrical characterization of the samples (sample C), we used a Vector Network Analyzer (VNA) connected to two rectangular X-band (*i.e.*, 8.2–12.4 GHz) waveguides through coaxial cables. This way, we could guarantee the precise measurement of the high-frequency scattering (S) parameters in terms of reflection (S_11_ and S_22_) and transmission (S_21_ and S_12_) coefficients between port 1 and port 2. However, to assess potential modifications of the sample's electrical characteristics under the application of an external DC bias voltage, we modified the setup by adding in between the two abovementioned waveguides the structure shown in Fig. 9. It consists of a metallic transition with the central hole (“Sample housing”) with the same horizontal and vertical dimensions as the aperture of the WR-90 X-band waveguides, *i.e.*, 22.86 mm × 11.43 mm, whereas the thickness is 1 cm (hence, much smaller than the wavelength at the maximum frequency of 12.4 GHz). At the top of this transition, two holes allow the insertion of two thin coaxial cables, whose inner conductors are exposed on the inside to solder them to the device under test (DUT). The position of these coaxial cables and the length of the inner conductors that protrude out of the transition toward the inside were optimized by electromagnetic (EM) simulations using CST Microwave Studio, to minimize their effect on the EM field during measurements, as will be shown later.

**Fig. 10:**
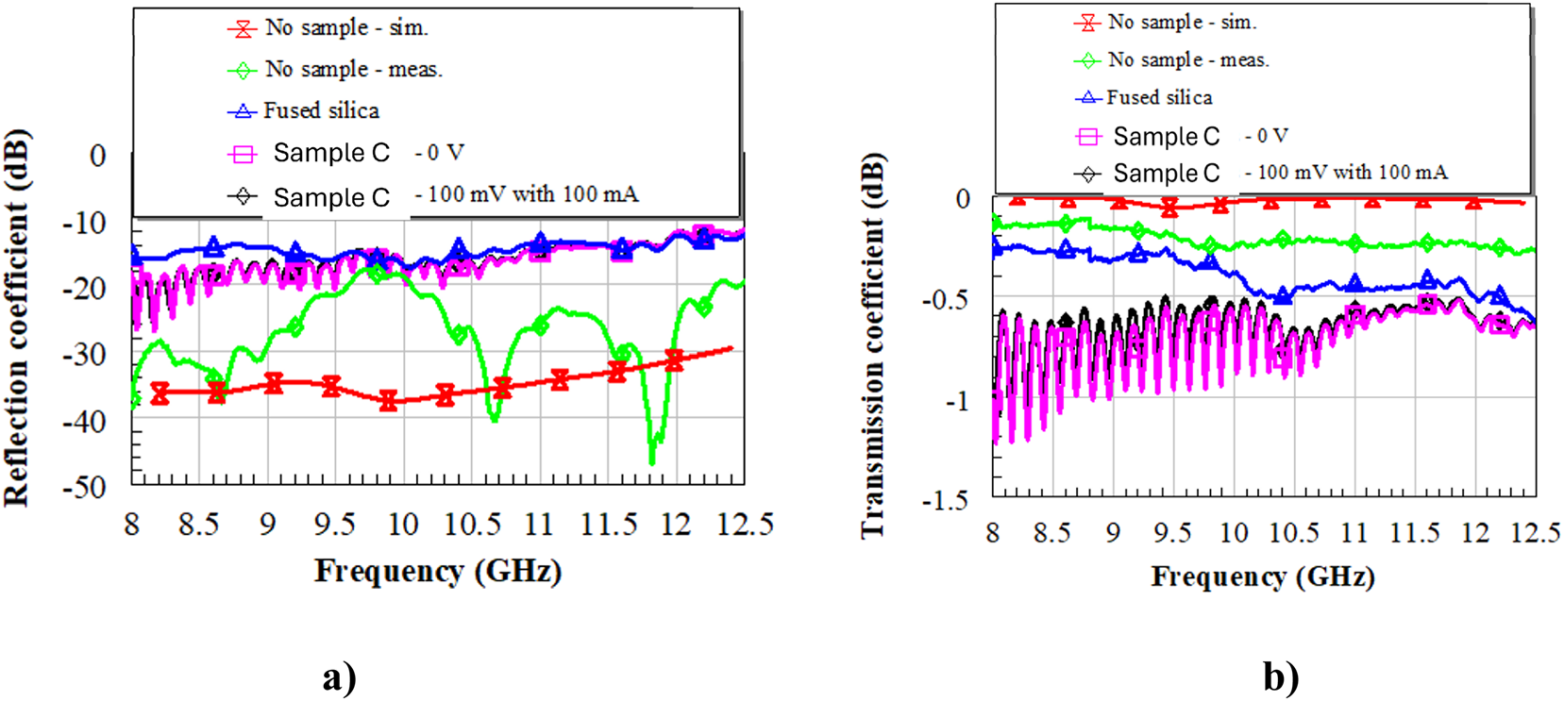
Simulated and measured *S* parameters with no sample inside the waveguides (red and green lines), and measured *S* parameters with samples of pure fused silica (blue line), fused silica with the as-deposited sample C in the unbiased state (pink line), and fused silica with the as-deposited sample C in the biased state (black line). (a) Reflection coefficient; (b) transmission coefficient. The frequency range of interest is the X band.

First, the setup was calibrated without any sample inside the waveguides (Fig. 10, red and green lines for the simulated and measured *S* parameters, respectively). One can notice that the measured reflection coefficient (Fig. 10a) is better than −20 dB all over the band of interest, whereas the transmission coefficient (Fig. 10b) is between −0.2 and −0.3 dB. This means that the effect of the inner conductors (Fig. 9) on the EM field inside the waveguides is quite negligible, which is a significant result in view of the successive characterizations. Then, we inserted a reference DUT of fused silica (blue line in Fig. 10) in the metallic transition and registered the measured *S* parameters. Since fused silica has a low dielectric constant (3.8 at 10 GHz) and low dielectric losses (loss tangent tan *δ* = 4 × 10^−4^ at 30 GHz), the reflection coefficient is better than 10 dB over the whole X band, with transmission as low as −0.5 dB at the upper part of the X band. After that, we measured the same samples of fused silica with the as-deposited sample C (this was made possible by glueing the inner conductors to the DUT using colloidal silver at room temperature, to not induce any phase transition inside the liquid crystals) at 0 V and with 100 mV of applied DC bias voltage (pink and black solid lines in Fig. 10, respectively). One can notice that the reflection coefficient is still good, even better than that of pure fused silica up to about 11.5 GHz, but the transmission coefficient worsens, with values attaining the range between −0.5 and −1.25 dB, meaning that the losses increase. Most interestingly, there are no significant differences between the unbiased (“0 V”) and biased (“100 mV with 100 mA”) cases.

**Fig. 11:**
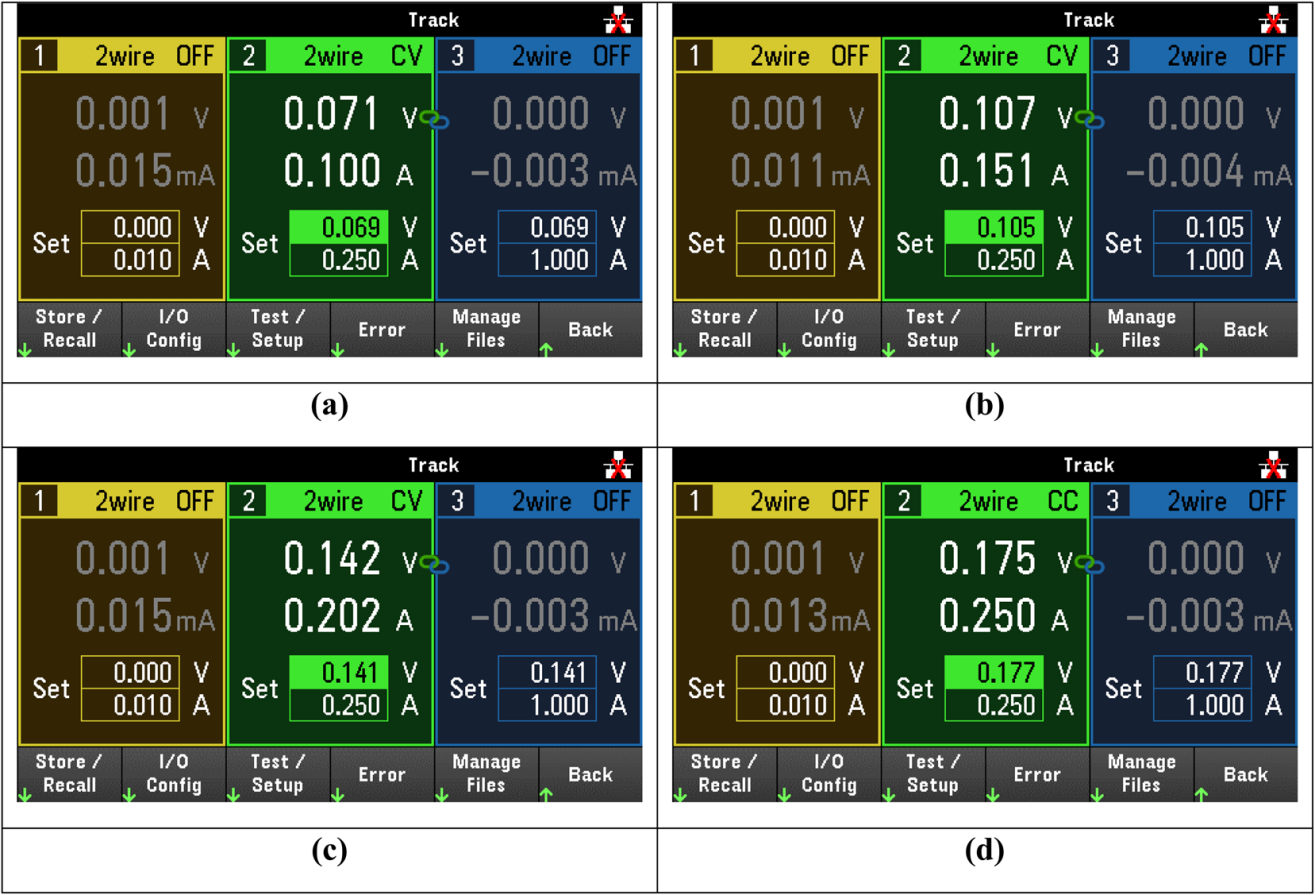
Screenshots of the voltage source with different voltage–current combinations applied to the sample of fused silica with sample C. From (a) to (d), the resistance slowly decreases from 0.71 to 0.7 Ω.

After applying an external DC bias voltage, we observed that only at 15 V a current started to flow between the inner conductors. Then, lowering the DC bias voltage to 1.5 V, the current level saturated. This is evident in Fig. 11, where we show different voltage–current combinations corresponding to resistance values between 0.7 and 0.71 Ω.

From the point of view of the microwave signals, as stated before we did not observe any relevant change. The only possible explanation is that a thin, geometrically confined conductive path was created between the tips of the inner conductors and inside the sample C layer, with an approximately constant resistance lower than 1 Ω.

It is worth mentioning that for samples A and B respectively we did not observe any electrical or electromagnetic shielding properties. Therefore, the electrical properties of these two polymeric films were not included in the previous results. Only the properties of sample C, which exhibited electromagnetic shielding properties, were highlighted. This can be attributed to the higher CLB content in sample C compared to samples A and B. Thus, increasing the CLB concentration enhanced the EMI shielding properties of the CLB-based polymeric films.

## 4. Conclusions

The present study highlights for the first time to our knowledge the synthesis and characterization of some nanocomposite materials obtained from polymers and CLB liquid crystals. The materials were synthesized by emulsion polymerization in the absence of the emulsifier, using different amounts of CLB. It is found that the amount of CLB used influences the morphology of the final particles.

Thus, although the synthesized particles have appropriate dimensions, demonstrated by SEM and DLS, in the case of the smallest amount of CLB used, the liquid crystal does not seem to be detectable by the XRD and DSC analysis methods. Through XPS analysis it was demonstrated that different morphologies were obtained, core–shell in the case of sample B and encapsulated in the case of samples A and C. These observations are also supported by the TEM images. The samples' electrical characteristics were evaluated under the application of an external DC bias voltage. After applying a DC bias voltage, current started flowing between the inner conductors at 15 V, saturating when the voltage was lowered to 1.5 V. Despite this, no significant changes were observed in microwave signals. A plausible scenario involves the creation of a confined, geometrically restricted conductive pathway between the inner conductor tips and within the layer of sample C, sustaining a consistently low resistance below 1 Ω.

In conclusion, the synthesis and characterization of different morphologies derived from polymers and CLB were highlighted.

## Conflicts of interest

There are no conflicts to declare.

## Data Availability

The data for this article are available at including synthesis and characterization of CLB-modified particles.
